# Randomised multiple centre trial of conservative versus liberal fluid administration for children receiving a kidney transplant (LIMITS): clinical trial protocol

**DOI:** 10.1136/bmjopen-2026-119384

**Published:** 2026-06-10

**Authors:** Nuala D M Calder, Fotini Kaloyirou, James Griffiths, Rosie Brown, Cara Hudson, Rupa Sharma, Hayley Hardwick, Lousie Oni, Chris Callaghan, Matt Stevenson, Mohan Shenoy, Ben Reynolds, Stephen Marks, Jo Wray, Helen Thomas, Mark J Peters, Wesley Hayes

**Affiliations:** 1 Centre for Bladder and Kidney Health, University College London, London, UK; 2 NHS Blood and Transplant Clinical Trials Unit, Cambridge, UK; 3 Department of Women's and Children’s Health, University of Liverpool, Liverpool, UK; 4 Department of Nephrology and Transplantation, Guy’s Hospital, London, UK; 5 School of Health and Health Related Research, University of Sheffield School of Health and Related Research, Sheffield, UK; 6 Department of Nephrology, Royal Manchester Children’s Hospital, Manchester, UK; 7 Department of Nephrology, Royal Hospital for Children, Glasgow, UK; 8 Department of Paediatric Nephrology, Great Ormond Street Hospital For Children NHS Trust, London, UK; 9 NIHR Biomedical Research Centre, University College London Great Ormond Street Institute of Child Health, London, UK; 10 Department of Psychology, Great Ormond Street Hospital for Children NHS Foundation Trust, London, UK; 11 Paediatric Intensive Care Unit, Great Ormond Street Hospital For Children NHS Trust, London, UK; 12 Department of Nephrology, Zurich Childrens Hospital, Zurich, Switzerland

**Keywords:** Paediatric transplant surgery, Paediatric nephrology, Fluid Therapy, HEALTH ECONOMICS, Renal transplantation

## Abstract

**Introduction:**

In current practice, fluid volumes administered to children following kidney transplant vary widely. Up to 52% of children experience fluid overload-related complications. Current fluid guidelines are not evidence-based and the optimal amount of fluid for children after transplant is not known. The aim of Randomised multiple centre trial of conservative versus LIberal fluid adMInisTration for children receiving a kidney tranSplant (LIMITS) is to determine whether relative limitation of fluid volume administered to children receiving kidney transplants is superior to liberal fluid volume administration.

**Methods and analysis:**

LIMITS is a pragmatic, open-label, UK-based, multicentre randomised controlled trial, with an internal pilot phase and integrated economic evaluation. A total of 140 children receiving kidney transplants will be randomised to receive either conservative postoperative fluid administration (maximum of 150 mL/m^2^/hour for no longer than 18 hours, followed by a fixed daily target of maximum 1.5 L/m^2^/day thereafter) versus the comparator of liberal postoperative fluid administration (fluid volume administered to replace urine output and insensible losses for at least 48 hours with target urine output >2 mL/kg/hour). The primary outcome is mean days at home in the first 30 days after kidney transplant. The primary outcome will be analysed using a mixed linear regression model adjusted for donor type (living vs deceased donor) and participant weight (<20 kg and ≥20 kg pretransplant) as fixed effects and transplant centre as a random effect. Cost-effectiveness will also be evaluated.

**Ethics and dissemination:**

The trial received Health Research Authority approval on 20 August 2025 (REC reference: 25/EE/0161, IRAS project ID: 354370). Findings will be presented to academic groups via national and international conferences and peer-reviewed journals. The patient and public involvement group will play an important part in disseminating the study findings to the public domain.

**Trial registration number:**

ISRCTN21516608.

**STRENGTHS AND LIMITATIONS OF THIS STUDY**

- This is the first randomised controlled trial to evaluate optimal fluid volume management following paediatric kidney transplantation.
- A tailored Hospital Stay Experience Questionnaire will assess participants’ and families’ experience of kidney transplantation.
- Variation in current clinical practice between centres and clinicians makes standardisation of intraoperative fluid volumes unfeasible.
- Blinding was not considered feasible as the randomisation arm will be evident from fluid volumes administered.

## Introduction

 Kidney transplantation is the treatment of choice for children with end-stage kidney disease. Large volumes of fluid are routinely administered to children following kidney transplant. As a result, complications of fluid overload are frequent.^1^

Excess fluid can cause electrolyte imbalances such as hyponatraemia, which has resulted in reports of cerebral oedema, seizures and even death in severe cases.^2 3^ Fluid overload after transplant is associated with pulmonary oedema, hypertension, delayed wound healing, increased blood transfusions, delayed discharge from hospital.^4 5^ Fluid overload prolongs hospital admission which impacts children’s kidney dialysis and transplant care capacity and increases healthcare costs.^6^

Observational data in paediatric transplant recipients <20 kg and adult kidney transplant recipients have shown favourable outcomes with conservative fluid volume administration.^7 8^ Clinical trial outcomes in adults with sepsis support the non-inferiority of a restrictive fluid strategy compared with previous liberal fluid volumes.^9^ In adult kidney transplant recipients, the traditional practice of large volume fluid administration is no longer recommended^10^ as large fluid volumes do not reduce the proportion of adult grafts that fail or are slow to function as previously thought.^11^

This change in adult transplant practice has not been widely adopted for children despite frequent fluid overload related complications because of a view that the risks and benefits may differ in smaller children.^1^ Central to this is concern about delayed transplant function and/or thrombosis with using a conservative fluid approach. Existing data demonstrate a low risk of transplant thrombosis. One year graft survival in paediatric kidneys is 97% (deceased donor transplant) and 98% (living donor transplant), so the overall risk of graft thrombosis is <3% nationally as vessel thrombosis typically leads to graft loss.^12^

The aim of the LIMITS trial is to determine if relative limitation of fluid volume administered to children receiving kidney transplant is superior to liberal fluid volume administration with a primary outcome of mean days at home up to 30 days after transplant.

## Methods and analysis

### Study design and setting

LIMITS is a two-arm pragmatic, open-label randomised controlled trial comparing relative limitation of fluid volume administered (intervention) to liberal fluid volume administration (comparator) in paediatric kidney transplant recipients across 10 UK paediatric kidney transplant centres and three patient identification centres (box 1). The study started on 1 January 2025, with a projected completion of 31 December 2027. The first patient was recruited on 23 October 2025.

Box 1 **Participating UK paediatric kidney transplant centres**

**Hospital**

Birmingham Children’s Hospital

Bristol Royal Hospital for Children

Evelina London Children’s Hospital

Great North Children’s Hospital

Great Ormond Street Hospital

Leeds Children’s Hospital

Nottingham Children’s Hospital

Royal Belfast Hospital for Sick Children

Royal Hospital for Children, Glasgow

Royal Manchester Children’s Hospital

Alder Hey Children’s Hospital*

University Hospital Southampton*

University Hospital of Wales*

*Participant identification centre.

### Intervention and comparator

The intervention is a conservative postoperative fluid administration approach: fluid volume is capped at a maximum of 150 mL/m^2^/hour for no longer than 18 hours following transplant, followed by a fixed daily target of maximum 1.5 L/m^2^/day thereafter.

The comparator is a liberal postoperative fluid approach: fluid volume is administered to replace urine output and insensible losses for at least 48 hours with target urine output >2 mL/kg/hour. Diuretics are permitted at any time. This is reflective of current majority practice in the UK.^1^

The intervention is based on physiological rationale, existing data on fluid volume administration post kidney transplant, and preparation work regarding acceptability. Prolonged use of a urine output replacement regimen can perpetuate a fluid overloaded state by counteracting the physiological response to fluid overload—diuresis. This can lead to a vicious cycle perpetuating polyuria and fluid overload. Capping the duration of the urine output replacement regimen may shorten the time period during which fluid overload post-transplant can occur. A time cap of 18 hours was chosen to facilitate change of fluid strategy on the morning following transplantation to reflect real world practice of clinical decision-making on morning ward rounds.

### Outcome measures

The primary outcome measure for clinical effectiveness is mean days at home in the first 30 days after kidney transplant. The primary outcome measure for cost-effectiveness is kidney related costs within the study period which encompasses all costs incurred and implications for patient health within the study.

Secondary outcome measures are as follows:

- Patient-reported experience of transplant hospital stay.
- Proportion of participants with systemic hypertension (systolic blood pressure above the 95th centile for age and height on 2 consecutive days) within 7 days after transplant.
- Proportion of participants with pulmonary oedema on chest X-ray within 7 days after transplant.
- Proportion of participants with severe acute hyponatraemia (defined as plasma sodium concentration <130 mmol/L) within 7 days after transplant.
- Proportion of participants receiving red blood cells within 7 days after transplant.
- Proportion of participants with transplant thrombosis in the postoperative period leading to graft failure within the first 30 days.
- Proportion of participants with delayed transplant function (dialysis within the first 7 days after transplant).
- Mean transplant function measured by estimated glomerular filtration rate (eGFR) at 3 months post-transplant.^13^

### Eligibility criteria

#### Inclusion criteria

- Children <18 years of age at the time of transplantation with valid informed consent.
- Children receiving a kidney only transplant from either a living or deceased donor, in a participating centre.

#### Exclusion criteria

- Multiorgan transplant recipients.

### Screening, recruitment and randomisation

All patients <18 years old awaiting kidney transplantation, from a living or deceased donor, will be screened for participation in participating sites plus three participant identification centres. The trial will be discussed by the clinical team during the pretransplant preparation period. The potential benefits and risks of study participation are included in age stratified study information sheets.

Under Clinical Trial Regulations, a person under the age of 16 years is deemed to be a ‘minor’ and an appropriate adult must give consent on behalf of the child. However, the wishes of the child must form part of the decision-making process and the child’s assent will be sought and recorded. For young people aged 16–18 years their informed consent will be taken (see onlinesupplemental files 1^4^ for copies of consent and assent forms). Any person aged 16 or over who lacks capacity to consent will not be considered for participation. Informed consent and patient assent will be taken by the Principal Investigator (PI) or delegated individual.

Participants will be randomised in a 1:1 ratio to the intervention and control groups via an online system (Sealed Envelope) which produced the randomisation list. Randomisation will be stratified by transplant centre and donor type (deceased vs living donation) and will further be balanced within blocks of varying, undisclosed sizes.

### Treatment

Participants randomised to the intervention arm will receive usual fluids with the volume administered capped to a maximum rate of 150 mL/m^2^/hour for no longer than 18 hours following transplant, then reduced to a fixed daily fluid target not exceeding 1.5 L/m^2^/day thereafter until day 5. No specific urine output is targeted. Diuretics should not be administered intraoperatively nor postoperatively.

Patients randomised to the comparator arm will receive usual fluids to replace urine output plus insensible losses for at least 48 hours following transplant. Target urine output will be >2 mL/kg/hour and diuretics can be given as per the clinical team’s usual practice.

**Figure 1:**
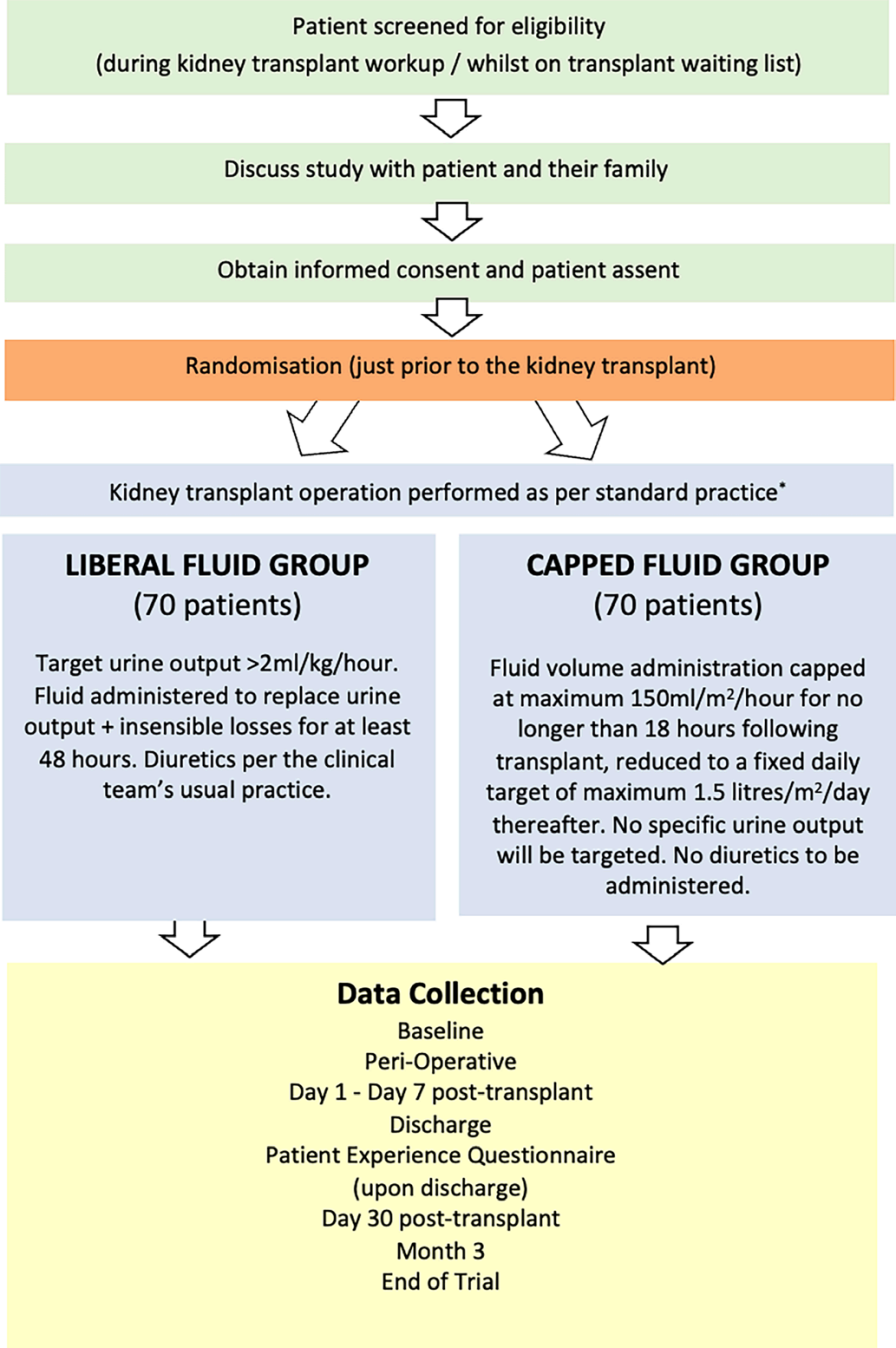
LIMITS study flow chart. *No diuretics to be given in the capped fluid group intraoperatively.

The choice of intravenous fluid (control or intervention) and decision to discontinue intravenous fluid and switch to enteral fluid will be determined by the treating clinician. All participants will receive routine clinical transplant care with no additional investigations or outpatient visits (figure 1, see onlinesupplemental files 1^4^ for schedule of procedures).

Each participant and their family have the right to withdraw from the trial at any time. The investigator may discontinue a participant from the trial at any time if considered necessary for any reason.

### Patient Reported Experience Measures: Hospital Stay Experience Questionnaire

A patient-reported experience questionnaire has been developed for this trial to better understand the patient and family experience of their transplant hospital admission. On the day of discharge the relevant version of the Hospital Stay Experience Questionnaire (HSEQ) will be completed anonymously by both the participants and their parent(s)/carer(s). Younger children or those with a learning disability may receive assistance with completion from their parent(s)/carer(s). For children under the age of 7, a parent/carer HSEQ only will be completed.

### Data collection

Data will be collected and entered directly by the site personnel into a validated electronic data capture system.

Data collection will include baseline demographics, details of end stage kidney disease and operation details including graft placement, cold ischaemia time and intraoperative fluid volume. In the post-transplant admission period (7 days) data collection will include fluid balance, weight, blood pressure, chest X-ray, oxygen use, red cell transfusion requirement, medication, sodium, dialysis requirement and graft function and survival. Data collection postdischarge but within the first 30 days post-transplant will include date of hospital discharge, details of readmissions and outpatient visits, transplant thrombosis leading to graft failure and graft function, plasma creatinine and height. The economic evaluation data will capture time spent in ward areas of varying acuity, return to theatre, additional investigations and long-term disability. In the event of a patient withdrawal information will be recorded detailing date, who initiated withdrawal and reason.

Data quality will be ensured through specific checks, query management, audit trails and source data verification. Role-based access control and system security measures will be used to protect data integrity and confidentiality. Archiving will be authorised by the Sponsor following submission of the end of study report. Essential documents will be retained for a minimum of 5 years after completion of the trial. Detailed data management procedures are described in the Data Management Plan.

### Statistical plan

#### Sample size

The sample size required is 140 participants randomised. The current mean number of ‘days at home up to 30 days after transplant’ is 16 days, with an SD of 7 days, as obtained from a retrospective analysis of 110 paediatric transplants from 3 centres in 2020–2021. Following review of published data in adult patients, and a consensus reached from the LIMITS patient and public involvement (PPI) group and the Young Persons Advisory Group (YPAG), the study is powered to detect a clinically meaningful difference of 3 days in the number of ‘days at home up to 30 days after transplant’ as this represents an important difference for patients.^14^

Due to the skewed nature of the data, the outcome was log-transformed, and the sample size was calculated on that scale to mirror the final analysis. A two-sided test, with 90% power, 5% type I error, 1:1 allocation, and allowing for two formal interim analyses at 50% and 75% for benefit and harm using non-binding O’Brien-Fleming boundaries, would require 126 participants. After allowing for 10% dropout, for example, if a transplant is unable to proceed following randomisation or the primary outcome cannot be obtained, the total number of participants required is 140.

### Interim analysis

A group sequential design with non-binding O’Brien-Fleming boundaries will inform early stopping of the trial in the case of strong evidence of harm or benefit, while preserving the overall 5% type I error rate. Two interim analyses will be conducted at 50% and 75% recruitment (when 70 and 105 participants have primary outcome data respectively). The independent Data Monitoring Committee (DMC) will review these to inform continuation of the trial alongside their wider review of the safety and integrity of the trial. In addition, the DMC will review serious adverse events (SAEs) at 20% recruitment (28 participants, randomised), in particular delayed transplant function and thrombosis.

### Analysis

The analyses will be described in detail in a full statistical analysis plan. The population used for efficacy analyses will be a modified intention-to-treat analysis which will include all randomised patients who receive a transplant, as although unlikely, it would be illogical to include any participant who did not receive a transplant after being randomised. This will be the primary analysis for the trial, though the primary outcome will also be analysed per protocol, as a sensitivity analysis.

### Primary outcome

The primary outcome, days at home in the first 30 days, will be analysed using a mixed linear regression model. Due to the skewed nature of the data, and to align with the methods used to generate the sample size, prior to the regression model being fitted, a log-transformation will be applied. The model will adjust for donor type (living vs deceased donor) and participant weight (<20 kg and >20 kg pretransplant) as fixed effects, and transplant centre as a random effect.

Sensitivity and subgroup analyses of the primary outcome will be performed using the same model as for the primary outcome, with an additional interaction term for each of the trial arm by subgroup categories. The following subgroup analyses are planned to assess whether the treatment effect differs between these subgroups:

- Donor type (living vs deceased donor): It is anticipated that days at home in the first 30 days post-transplant will be fewer for deceased donor kidney recipients.
- Participant wt (<20 kg and >20 kg pretransplant): It is anticipated that the effect size will be greater for participants <20 kg.

### Secondary outcomes

Secondary outcomes will be analysed using mixed logistic or linear regression models as appropriate with the same risk-adjustment as for the primary outcome. For transplant function at 3 months post-transplant measured by eGFR, transformations will be applied prior to modelling, if appropriate.

Any missing primary and secondary outcome data will be summarised. Primary and secondary outcome measures will not be imputed and these will be treated as missing data and excluded from the relevant analyses. If outcome data are missing for more than 25% of participants, outcomes will not be reported.

To explore if missing values have an undue impact on the primary outcome result, a sensitivity analysis using multiple imputation will be performed if the primary outcome is missing in more than 5% of the participants included in the modified intention-to-treat analysis.

### Cost-effectiveness outcomes

The primary outcome for the cost-effectiveness analysis will be kidney-related costs within the study period. The analysis will be confined to the data observed within the study with all costs incurred and implications for patient health considered.

To supplement the primary economic analyses, a secondary analysis will be conducted that does not assume that efficacy is identical in both arms. This analysis will provide an estimate of the cost for a unit eGFR improvement at 3 months. This will allow clinicians to make judgements on whether any increased costs are justified by the clinical benefit provided.

### Patient experience of hospital stay data

Questionnaires will be analysed using appropriate descriptive and non-parametric statistics.

### Patient and public involvement

A kidney transplant patient and his mother are co-applicants for the grant and members of the Trial Steering Committee (TSC). They make sure that the trial remains patient-centred throughout its delivery and will lead the dissemination of results to patients and families.

The research plan was developed with the PPI group which comprised five parents of children who had recently undergone kidney transplant and two teenage kidney transplant patients. Feedback was also sought from the YPAG at Great Ormond Street Hospital. The PPI group and YPAG highlighted aspects and complications that were important to them which helped formulate outcome measures and develop the study protocol.

There was significant patient and family input into the development of the two HSEQ. Final versions were piloted with a small group of kidney transplant patients and their parents.

### Ethics, safety and dissemination

#### Ethical compliance

The Investigator will ensure that this trial is conducted in accordance with the principles of the Declaration of Helsinki, ICH Good Clinical Practice Guidelines and in accordance with the terms and conditions of the ethical approval given to the trial. A favourable opinion has been given by a Research Ethics Committee (IRAS project ID: 354370, REC reference: 25/EE/0161). Important protocol modifications will be communicated to all relevant parties in writing with relevant updated Protocol and other trial documentation as relevant. The Research Ethics Committee that reviewed the study was the East of England–Cambridge South Research Ethics Committee.

### Safety

#### Adverse events and expected adverse drug reactions

Non-SAEs will not be reported. SAEs will be assessed by delegated individuals and the clinical trials unit (CTU) notified within 24 hours. SAEs will be monitored regularly by the DMC to ensure ongoing safety of trial participants. All SAEs will be followed up until resolution or stabilisation. All deaths will be reported as an SAE.

Certain SAEs are anticipated in this patient group and have therefore been excluded from trial safety reporting. These include surgical complications, infective complications, immunological complications and medication-related complications.

### Trial oversight

The Trial Management Group (TMG) will be responsible for the day-to-day running and management of the trial.

The TSC will meet annually and provide overall supervision for the trial and provide advice through its independent Chair. The ultimate decision on continuation of the trial lies with the TSC.

The DMC will be responsible for safety monitoring. Protocol deviations will be reported to the DMC. Recommendations made by the DMC will be forwarded to the TSC for a final decision. The Sponsor or delegate will forward such decisions to the regulatory authorities as appropriate. The DMC advises on the statistical analysis plan but otherwise will be independent of the study team and will have no direct involvement in other aspects of the trial.

#### Monitoring compliance

Non-compliance is defined as any participant in the intervention arm receiving in excess of 4700 mL/m^2^ fluid in the first 48 hours post-transplant or any child in the comparator arm receiving less fluid than urine output replacement in the first 48 hours post-transplant. Non-compliance cases will be recorded as protocol deviations. Separation between fluid volumes received by participants in the study arms will be monitored to ensure that a minimum median difference in fluid volume administered in the first 72 hours of 1.5 L/m^2^ is maintained throughout the trial at each site. Adherence to the capped and liberal fluid arms will be monitored by the CTU and will be reported to the TMG and DMC on a regular basis. If significant non-compliance were to occur, further site-specific training would be undertaken, with closer subsequent monitoring. A fluid calculator tool is provided to sites to help guide fluid volumes and monitor self-compliance.

### Dissemination

The final study data set will be analysed and results published as soon as possible following completion of study follow-up, final data checks and database lock. The TMG will form the basis of the Writing Committee and will advise on the nature of publications, with the TSC’s input.

Study findings will be presented to academic and non-academic groups. The PPI group will play an important part in disseminating the study findings into the public domain. Dissemination to non-academic audiences including service users, commissioners, clinicians and service providers will be facilitated through the use of existing networks, that is, email lists and social media.

All research teams and PPI members involved in the study will be invited to a close-out meeting to discuss the findings of the study.

Open access, peer-reviewed academic outputs and research reports together with associated summaries and key findings will be produced for funders, policy makers and NHS audiences and held on the study website.

Any publications arising from this study will adhere to the National Institute for Health and Care Research funding and support outputs guidance.

## Supplementary material

10.1136/bmjopen-2026-119384 online supplemental file 1

10.1136/bmjopen-2026-119384 online supplemental file 2

10.1136/bmjopen-2026-119384 online supplemental file 3

10.1136/bmjopen-2026-119384 online supplemental file 4
