# Supplementary material for: Randomised multiple centre trial of conservative versus liberal fluid administration for children receiving a kidney transplant (LIMITS): clinical trial protocol
Source: BMJ Open. 2026 Jun 10;16(6):e119384. doi: 10.1136/bmjopen-2026-119384 (PMC13264947; doi:10.1136/bmjopen-2026-119384)
Supplement: online supplemental file 2 [file bmjopen-16-6-s002.docx]

**Schedule of Procedures**

|  | **Screening** | **Baseline (Pre-Tx)** | | **Day of Tx (Day 0)** | | | **Day 1 Post-Tx** | **Day 2 Post-Tx** | **Day3-5 Post-Tx** | **Day 6 Post-Tx** | | | **Day 6-7 Post-Tx** | | **Hospital Discharge** | **30 days Post-Tx** | | **Month 3 Post-Tx** | |
| --- | --- | --- | --- | --- | --- | --- | --- | --- | --- | --- | --- | --- | --- | --- | --- | --- | --- | --- | --- |
| ENROLMENT | | | | | | | | | | | | | | | | | | | |
| Eligibility assessment | **X** | |  | | **X** |  | |  |  | |  |  | |  | | |  | |  |
| Informed consent |  | | **X** | |  |  | |  |  | |  |  | |  | | |  | |  |
| Baseline Demographics |  | | **X** | |  |  | |  |  | |  |  | |  | | |  | |  |
| Baseline (kidney disease details) |  | | **X** | |  |  | |  |  | |  |  | |  | | |  | |  |
| Re-confirm consent/assent |  | |  | | **X** |  | |  |  | |  |  | |  | | |  | |  |
| Randomisation |  | |  | | **X** |  | |  |  | |  |  | |  | | |  | |  |
| **TREATMENT ARMS** | | | | | | | | | | | | | | | | | | | |
| Administration of trial fluid volume – capped fluid administration* |  | |  | | **X** | **X** | | **X** | **X** | | **(X)** | **(X)** | | **(X)** | | |  | |  |
| Administration of trial fluid volume – liberal fluid administration ** |  | |  | | **X** | **X** | | **X** | **X** | | **(X)** | **(X)** | | **(X)** | | |  | |  |
| **DATA COLLECTION** | | | | | | | | | | | | | | | | | | | |
| Transplant Operation Data |  | |  | | **X** |  | |  |  | |  |  | |  | | |  | |  |
| Height  (data collection from medical record) |  | | **X** | |  |  | |  |  | |  |  | |  | | |  | | **X** |
| Weight & Blood pressure  (data collection from medical record) |  | | **X** | | **X** | **X** | | **X** | **X** | | **X** | **X** | |  | | |  | |  |
| Graft Function  (data collection from medical record) |  | |  | | **X** | **X** | | **X** | **X** | | **X** | **X** | |  | | | **X** | |  |
| Fluid Input & Output |  | |  | | 5 days from arrival in recovery | | | | | |  |  | |  | | |  | |  |
| Intraoperative total fluid input (volume) |  | |  | | **X** |  | |  |  | |  |  | |  | | |  | |  |
| Blood test results (lowest Na+), O2 administration (Y/N) (data collection from medical record) |  | |  | | **X** | **X** | | **X** | **X** | | **X** | **X** | |  | | |  | |  |
| Medications and Blood products (data collection from medical record) |  | |  | | **X** | **X** | | **X** | **X** | | **X** | **X** | |  | | |  | |  |
| Transplant thrombosis |  | |  | | **X** | **X** | | **X** | **X** | | **X** | **X** | |  | | | **X** | |  |
| Safety Reporting |  | |  | | **X** | **X** | | **X** | **X** | | **X** | **X** | | **X** | | |  | |  |
| Patient Experience of Hospital Stay Questionnaire |  | |  | |  |  | |  |  | |  |  | | **X** | | |  | |  |
| Discharge Date |  | |  | |  |  | |  |  | |  |  | | **X** | | |  | |  |
| **FOLLOW UP** | | | | | | | | | | | | | | | | | | | |
| Re-admission |  | |  | |  |  | |  |  | |  |  | |  | | | **X** | |  |
| No. of days spent at home up to 30 days after the day of transplant *** |  | |  | |  |  | |  |  | |  |  | |  | | | **X** | |  |
| No of days of outpatient hospital visits |  | |  | |  |  | |  |  | |  |  | |  | | | **X** | |  |
| Plasma creatinine (data collection from routine blood tests and medical record) |  | |  | |  |  | |  |  | |  |  | |  | | |  | | **X** |

^*^Fluid volume administration capped at maximum 150ml/m^2^/hour for no longer than 18 hours following arrival in recovery, reduced to a fixed daily target of maximum 1.5 litres/m^2^/day thereafter. No specific urine output will be targeted. No diuretics to be administered during the transplant and post-transplant.

^**^ Target urine output >2ml/kg/hour. Fluid administered to replace urine output + insensible losses for at least 48 hours following arrival in recovery. Diuretics per the clinical team’s usual practice.

^***^ Day of discharge to home or to patient hotel will be included.

(X) fluid data will not be collected beyond Day 5.
