# Supplementary material for: Randomised multiple centre trial of conservative versus liberal fluid administration for children receiving a kidney transplant (LIMITS): clinical trial protocol
Source: BMJ Open. 2026 Jun 10;16(6):e119384. doi: 10.1136/bmjopen-2026-119384 (PMC13264947; doi:10.1136/bmjopen-2026-119384)
Supplement: online supplemental file 3 [file bmjopen-16-6-s003.pdf]

A Randomised Multiple Centre Trial of Conservative versus Liberal Fluid administration for Children Receiving a Kidney Transplant (LIMITS)

Chief Investigator: Dr Wesley Hayes

# ASSENT FORM

| | | | |
|---|---|---|---|
| Participating Site (Site Code): | | | |
| Principal Investigator: | | | |
| Participant Name: | | Date of Birth: | |
| Screening number: | SCR - ☐☐☐ - ☐☐☐ - ☐☐☐☐☐ | | |
| Trial ID (if randomised): | R☐☐☐ - ☐☐☐ | | |

Please tick each box you agree

| | | |
|---|---|---|
| 1 | Has somebody else explained this research study to you? | ☐ |
| 2 | Do you understand what this study is about? | ☐ |
| 3 | Have you asked all the questions you want, and have they been answered in a way you understand? | ☐ |
| 4 | Do you understand it’s OK to stop taking part at any time? | ☐ |
| 5 | Are you happy to take part? | ☐ |

If you do want to take part, you can write your name below:

Your name ___________________________________________

Date ___________________________________________

The doctor who explained this study to you needs to sign too:

Print Name ___________________________________________

Sign ___________________________________________

Date ___________________________________________

___________________________________

Once the assent form has been signed by both parties the participant / family should receive a copy of the signed and dated form. A copy must be enclosed in the participant's medical record and the original signed and dated consent form should be kept with the project's main documents, which must be kept in a secure location.
