# Supplementary material for: Randomised multiple centre trial of conservative versus liberal fluid administration for children receiving a kidney transplant (LIMITS): clinical trial protocol
Source: BMJ Open. 2026 Jun 10;16(6):e119384. doi: 10.1136/bmjopen-2026-119384 (PMC13264947; doi:10.1136/bmjopen-2026-119384)
Supplement: online supplemental file 4 [file bmjopen-16-6-s004.pdf]

A Randomised Multiple Centre Trial of Conservative versus Liberal Fluid administration for Children Receiving a Kidney Transplant (LIMITS)

Chief Investigator: Dr Wesley Hayes

CONSENT FORM (PARENT / GUARDIAN)

Participating Site (Site Code):			
Principal Investigator:			
Participant Name:		Date of Birth:	
Screening number:	SCR - ☐☐☐ - ☐☐☐ - ☐☐☐☐☐		
Trial ID (if randomised):	R☐☐☐ - ☐☐☐		

Please **initial** each box if you agree

1	I confirm that I have read and understood the LIMITS Parent / Guardian Information Sheet (version .... dated ....) I have had the opportunity to consider the information, ask questions and have had these answered satisfactorily.	☐
2	I understand that participation is voluntary and that I am free to withdraw my child at any time, without giving any reason, and without their medical care or legal rights being affected. All data accrued prior to withdrawal will be collected and used in the analysis.	☐
3	I understand that relevant sections of my child's medical notes and data collected during the study may be looked at by the staff from the study team or the Sponsor / NHS Blood and Transplant CTU for monitoring, audit purposes, where relevant to this research. I give permission for these individuals to have access to their records.	☐
4	I agree that my child's General Practitioner (GP) will be given information about their participation in this study.	☐
5	I understand that all my child's data will be anonymised and that information will remain confidential.	☐
6	I understand that information about my child's kidney transplant, which is already provided to NHS Blood and Transplant, may be used for this study.	☐

7	I understand that the information collected about my child could also be used to support other ethically approved research or research funding applications in the future. I give permission for this to be shared anonymously with other researchers (optional).	☐
8	I understand that the results of this study will be published anonymously and will not include any identifying characteristics.	☐
9	I agree to my child's participation in the above study.	☐

Informed consent provided remotely? YES ☐ NO ☐

PRINT NAME	DATE	TIME	SIGNATURE

RELATIONSHIP TO PATIENT: ______________________

NAME OF PERSON TAKING CONSENT	DATE	SIGNATURE

If consent is provided remotely, please return the signed consent form by post or e-mail to the address below:

Postal address (if returning by post):

E-mail address (if returning by email):

____

Once consent form has been signed by both parties the participant / family should receive a copy of the signed and dated consent form. A copy must be enclosed in the participant's medical record and the original signed and dated consent form should be kept with the project's main documents, which must be kept in a secure location.
