# Supplementary material for: Randomised multiple centre trial of conservative versus liberal fluid administration for children receiving a kidney transplant (LIMITS): clinical trial protocol
Source: BMJ Open. 2026 Jun 10;16(6):e119384. doi: 10.1136/bmjopen-2026-119384 (PMC13264947; doi:10.1136/bmjopen-2026-119384)
Supplement: Supplementary file 5 [file bmjopen-2026-119384.reviewer_comments.pdf]

PEER REVIEW HISTORY

BMJ Open publishes all reviews undertaken for accepted manuscripts. Reviewers are asked to complete a checklist review form and are provided with free text boxes to elaborate on their assessment. These free text comments are reproduced below.

ARTICLE DETAILS

Title (Provisional)

Clinical Trial Protocol: A Randomised Multiple Centre Trial of Conservative versus Liberal Fluid administration for Children Receiving a Kidney Transplant (LIMITS)

Authors

Calder, Nuala D M; Kaloyirou, Fotini; Griffiths, James; Brown, Rosie; Hudson, Cara; Sharma, Rupa; Hardwick, Hayley; Oni, Lousie; Callaghan, Chris; Stevenson, Matt; Shenoy, Mohan; Reynolds, Ben; Marks, Dr. Stephen; Wray, Jo; Thomas, Helen; Peters, Mark J.; Hayes, Wesley

---

VERSION 1 - REVIEW

**Reviewer**	**1**
**Name**	**Trachtman, Howard**
**Affiliation**	**Division of Nephrology, Department of Pediatrics, University of Michigan, Ann Arbor, Michigan, USA**
**Date**	**13-Apr-2026**
**COI**	**None**

This protocol addresses an important clinical problem, and the study is well designed. The text is clear. The only gaps that I can identify are (1) a section that discusses how they will deal with clinical scenarios that might warrant changes in the fluid regimen such as significant changes in BP or bleeding; (2) it would be helpful to clarify how they will handle overlap between IV and PO fluids and the transition to sole PO intake and (3) the need to account for fluids given with medications

---

VERSION 1 - AUTHOR RESPONSE

Reviewer: 1

Dr. Howard Trachtman, Division of Nephrology, Department of Pediatrics, University of Michigan, Ann Arbor, Michigan, USA

Comments to the Author:
This protocol addresses an important clinical problem, and the study is well designed. The text is clear.

Many thanks for your comprehensive review of the manuscript and highlighting the below points which I will now address.

The only gaps that I can identify are (1) a section that discusses how they will deal with clinical scenarios that might warrant changes in the fluid regimen such as significant changes in BP or bleeding;

LIMITS is a pragmatic trial in which the aim of the intervention is to limit excess maintenance IV fluid administration. Clinical management is otherwise determined by the clinical paediatric nephrology team at each site. Given the pragmatic trial design, we accept that protocol deviations to manage exceptional situations such as bleeding or marked hypovolemia may occur. However, we do not expect the added variability from these infrequent situations to impact the primary analysis. We discuss clinical management of hypovolemia from fluid/blood loss at each of the site initiation visits and site specific training sessions, but chose not to specify this in the trial protocol in order to allow Investigators freedom to tailor the management of these scenarios in a participant specific manner.

(2) it would be helpful to clarify how they will handle overlap between IV and PO fluids and the transition to sole PO intake

The aim of the intervention is to limit excess IV fluid administration, however given the pragmatic nature of the trial we have not specified the route of fluid delivery as there may be specific individual participant reasons to delay switching from IV to enteral route (e.g. post-operative ileus). This decision is left to the discretion of study site investigators (clinical paediatric nephrology team) based on their clinical assessment of individual participants' status and clinical needs.

(3) the need to account for fluids given with medications

Data from preparatory work for the study confirmed that medication diluents do not constitute a significant proportion of the total fluid administered to participants. Given this, and the pragmatic trial design, we have not specified the need to account for medication diluents in the protocol.

Thank you again for your review of this manuscript. We look forward to hearing from you.

VERSION 2 - REVIEW

Reviewer 1

Name Trachtman, Howard

Affiliation Division of Nephrology, Department of Pediatrics, University of Michigan, Ann Arbor, Michigan, USA

Date 26-May-2026

COI

The paper is thoughtfully revised and is an excellent piece of work
